# Animal-Assisted Therapy in palliative care: a scoping review

**DOI:** 10.3389/fpsyg.2024.1478264

**Published:** 2024-12-05

**Authors:** Laura Palomino-Lázaro, María Rueda-Extremera, María Cantero-García

**Affiliations:** ^1^Psicología, Facultad de Ciencias de la Salud, Universidad Internacional de Valencia (VIU), Castelló de la Plana, Spain; ^2^Psicología, Facultad de Ciencias de la Salud, Universidad a Distancia de Madrid (UDIMA), Madrid, Spain

**Keywords:** palliative care, patients, quality of life, animal-assisted therapy (AAT), health

## Abstract

**Background:**

Animal-assisted therapy (AAT) is increasingly recognized as beneficial in palliative care, aiming to enhance the well-being of terminally ill patients. Palliative care focuses on holistic support for physical, emotional, social, and spiritual needs. AAT uses animal interactions to alleviate symptoms such as pain, anxiety, and depression, promoting social engagement and emotional comfort. This review assesses AAT’s effectiveness in enhancing the quality of life for palliative care recipients.

**Aim:**

Synthesizing literature on AAT in palliative care, the review examines its impact on physical symptoms, emotional well-being, social interactions, and overall comfort. By analyzing diverse studies, it aims to elucidate AAT’s therapeutic potential and identify research gaps.

**Design:**

Scoping review.

**Data sources:**

Searches in PubMed, ProQuest, Psychology Database, and Scopus identified relevant studies evaluating AAT interventions in palliative care. Data extraction focused on study characteristics, participant demographics, AAT interventions, and reported outcomes.

**Results:**

Studies consistently report positive outcomes of AAT in palliative care, including reduced pain, anxiety, depression, and improved mood and well-being. AAT also enhances social interactions and emotional support, albeit with variations in study designs.

**Conclusion:**

AAT holds promise for improving quality of life in palliative care by addressing physical, emotional, and social needs. Future research should standardize methodologies, explore mechanisms of action, and optimize AAT integration into comprehensive palliative care strategies.

## Introduction

Palliative Care is considered a right that individuals have to health (Gomes and Othero, 2016). Palliative Care is developed around three main characteristics: multidimensional assessment and management of distress (physical and emotional), interdisciplinary care involving multiple professionals in addition to physicians and nurses, and an emphasis on caring not only for patients but also their families (Lutz, 2011). The World Health Organization (WHO) defines Palliative Care as an approach to improve the quality of life for patients and families facing problems associated with life-threatening illnesses, through prevention and relief of suffering, early identification, impeccable assessment, and treatment of pain and other physical, psychosocial, and spiritual problems (World Health Organization, 2007).

Palliative Care has evolved from a philosophy of end-of-life care to a professional discipline encompassing symptom management, psychosocial and spiritual care, caregiver support, physician-patient communication, complex decision-making, and end-of-life issues (Jacobsen et al., 2011). Palliative Care should not be limited to the last days or weeks of life, as key aspects of care can and should be provided much earlier in the disease trajectory to enhance patient quality of life (Greer et al., 2013). Positioned to collaborate with oncology teams, Palliative Care addresses the complex supportive care needs of cancer patients and their families (Hannon et al., 2016), starting from the early stages of a gradual terminal illness (Sepúlveda, 2005).

The primary goal of Palliative Care is to improve the quality of life for patients and their families facing terminal illnesses (World Health Organization, 2020). This is best achieved through a multidisciplinary team approach, requiring effective and timely communication among primary, secondary, and tertiary healthcare providers. Key components include management of physical symptoms, psychosocial care, support for families and caregivers, and bereavement follow-up. While often associated with cancer care due to predictable symptom burden and disease trajectory, non-malignant advanced illnesses impose similar symptom burdens and care needs yet are less likely to access Palliative Care (Mounsey et al., 2018).

Evidence shows that for patients with serious illnesses, receiving Palliative Care is better in all aspects than not having access, and early access is better than late access (Hawley, 2017). Despite improving the quality of life for patients and families, Palliative Care services remain underutilized (Woo et al., 2011). Numerous barriers to accessing Palliative Care services have been identified, including patient and family reluctance, fear, misconceptions, ignorance, and lack of awareness of resources (Lillyman et al., 2011). Knowledge about Palliative Care could help overcome these fears and misunderstandings, thus improving their utilization through better understanding of their benefits, as evidenced by Kozlov et al. (2017), who reported that Palliative Care knowledge positively correlated with improved Palliative Care service utilization. Education on Palliative Care is crucial for increasing public knowledge and general awareness (Schim and Raspa, 2007).

The principles of Palliative Care (Pessini and Bertachini, 2006) emphasize achieving and maintaining maximum control of pain and symptom management. This requires an assessment of each sick individual, considering their history, physical examination, etc. They value life and perceive death as a normal phase. The purpose of Palliative Care is to ensure that sick individuals are empowered and encouraged to live their lives fully until death arrives. Palliative Care neither hastens nor postpones death. They incorporate psychological and spiritual aspects into patient care, provide support to encourage patients to live actively until death, and involve the family in addressing the patient’s illness and grief. Grief begins before the patient’s death. Palliative Care requires teamwork, with a core team comprising a physician, nurse, and social worker, and it is beneficial at the onset of the disease, integrating with life-prolonging therapies.

Early inclusion of Palliative Care is associated with improvements in quality of life, symptom burden, and satisfaction with care received (Toca, 2021). According to the National Institutes of Health (National Institute of Nursing Research (NIH), 2020), researchers have studied the positive effects of Palliative Care on patients and their families. Patients receiving Palliative Care report improvements in pain, nausea, and difficulty breathing; communication with healthcare professionals and family members; and emotional support. Early initiation of Palliative Care in the disease course ensures that care aligns more closely with patient wishes, reduces stress, enhances confidence in caregiving decisions, meets emotional and spiritual needs of patients and families. A study by Bakitas et al. (2009) demonstrated that integrating a nurse-led Palliative Care intervention concurrently with cancer treatments improved quality of life and reduced depressed mood. Similarly, findings from Triplett et al. (2017) highlight the benefits of early Palliative Care, including improved quality of life, mood, less aggressive end-of-life care, better survival, patient health, and caregiver satisfaction.

Animal-Assisted Therapy (AAT) is defined as a structured therapeutic intervention aimed at improving physical, cognitive, behavioral, and/or socioemotional functioning of individuals (Jegatheesan et al., 2018). This therapy, historically underdocumented until the late 20th century, has seen increased research and documentation of its effects on individuals (López-Fernández et al., 2024). Most commonly involving dogs, AAT has been used worldwide across various settings. Literature reviews generally support these therapies with favorable outcomes, yet more rigorous and standardized research is needed to strengthen existing evidence (Arriba de La Paz, 2022). Guidelines ensure both human and animal welfare in AAT. For human welfare, measures include safety precautions for recipients, awareness of specific allergies, high-risk populations, and exclusion criteria. Caregivers and professionals should understand recipients’ needs and beliefs regarding animals involved in interventions. For animal welfare, only domesticated animals are eligible, assessed for suitability by animal behavior experts, ensuring health, comfort, and adequate rest before and after sessions. Precautions against zoonoses are essential, requiring veterinary health checks for animals annually, including parasite prevention and detection of potentially zoonotic microorganisms (Jegatheesan et al., 2018).

Scientific studies on AAT, particularly with dogs, demonstrate acceptance by medical teams and document safety and efficacy in various clinical contexts (Silva and Osório, 2018). Designing an AAT program involves species characteristics, individual compatibility, therapeutic rationale for interacting with specific animals, considerations for animal welfare, and patient safety during interactions (Chitic et al., 2012). Research supports beneficial effects of animal interaction on human health, such as increased serotonin levels from petting dogs (Johnson et al., 2008), release of beneficial hormones like prolactin, oxytocin, and phenylethylamine, improved self-esteem and self-confidence (Boe, 2008), reduced irritability in mentally ill patients (Cabra, 2012), and enhanced psychological immunological response and comfort in terminal cancer patients (Müschel, Bibbo, 2013). Studies also show decreased depression and increased arterial oxygen levels during chemotherapy in adult cancer patients (Orlandi et al., 2007), improved mood and reduced agitation in geriatric patients (Perkins et al., 2008), and decreased anxiety and loneliness in long-term care settings (Banks and Banks, 2002).

### Objectives

1. To investigate the impact of AAT on the following aspects:
 o Physical symptoms: pain, nausea, fatigue, appetite, and sleep.
 o Psychological symptoms: anxiety, depression, fear, loneliness, and self-esteem.
 o Social symptoms: social support, quality of relationships, and communication.
 o Spiritual symptoms: inner peace, sense of life, and meaning of death.
2. To explore the perceptions of patients, families, and healthcare professionals regarding AAT in palliative care (Palliative Care).
3. To study the effectiveness and feasibility of AAT in improving the quality of life of patients in Palliative Care.

### Hypotheses

1. AAT will have a positive outcome on the quality of life of individuals in Palliative Care, including:
 o Reduction of physical symptoms (pain, nausea, fatigue, etc.).
 o Decrease in psychological symptoms (anxiety, depression, fear, etc.).
 o Progress in social symptoms (social support, quality of relationships, etc.).
 o Enhancement of spiritual symptoms (inner peace, sense of life, etc.).
2. AAT in Palliative Care is perceived as beneficial by patients, families, and healthcare professionals.
3. AAT implemented in Palliative Care settings is effective and feasible in improving the quality of life of patients, addressing both physical and emotional, social, and spiritual aspects.

## Method

Following the structure of the PICO questions, the following information is available:

- *P (Population):* Individuals in palliative care.
- *I (Intervention):* Animal-assisted therapy.
- *C (Comparison)*: No intervention.
- *(Outcomes)*: Impact of animal-assisted therapy on quality of life, emotional well-being, pain management, anxiety, and depression in individuals in palliative care.

The search was conducted from February 22nd to May 1st, 2024. It was performed in the following databases: PubMed, Proquest, Psychology Database, and Scopus. The descriptors used were: (palliative care) AND (animal-assisted therapy), and (effects of animal-assisted therapy) AND (palliative care); ((animal-assisted therapy) OR (animal-assisted intervention)) AND (palliative care); (animal-assisted therapy) OR (therapy animals) AND (end of life care) and (animal-assisted therapy) OR (therapy animals) AND (pain, anxiety and depression). These searches resulted in a total of 3,280 articles, which were further analyzed in detail according to the established criteria.

Inclusion and exclusion criteria for the articles: Inclusion criteria: (1) written in English, Spanish, or Portuguese, (2) related to animal-assisted therapy and palliative care, (3) focused on patients receiving palliative care, and (4) studies evaluating outcomes such as quality of life, pain, anxiety, depression, or general well-being. Exclusion criteria were: (1) evaluating other variables not specifically related to the topic of interest, (2) evaluating another general population, (3) published in languages other than those specified in the inclusion criteria, and (4) systematic review studies.

**Figure 1:**
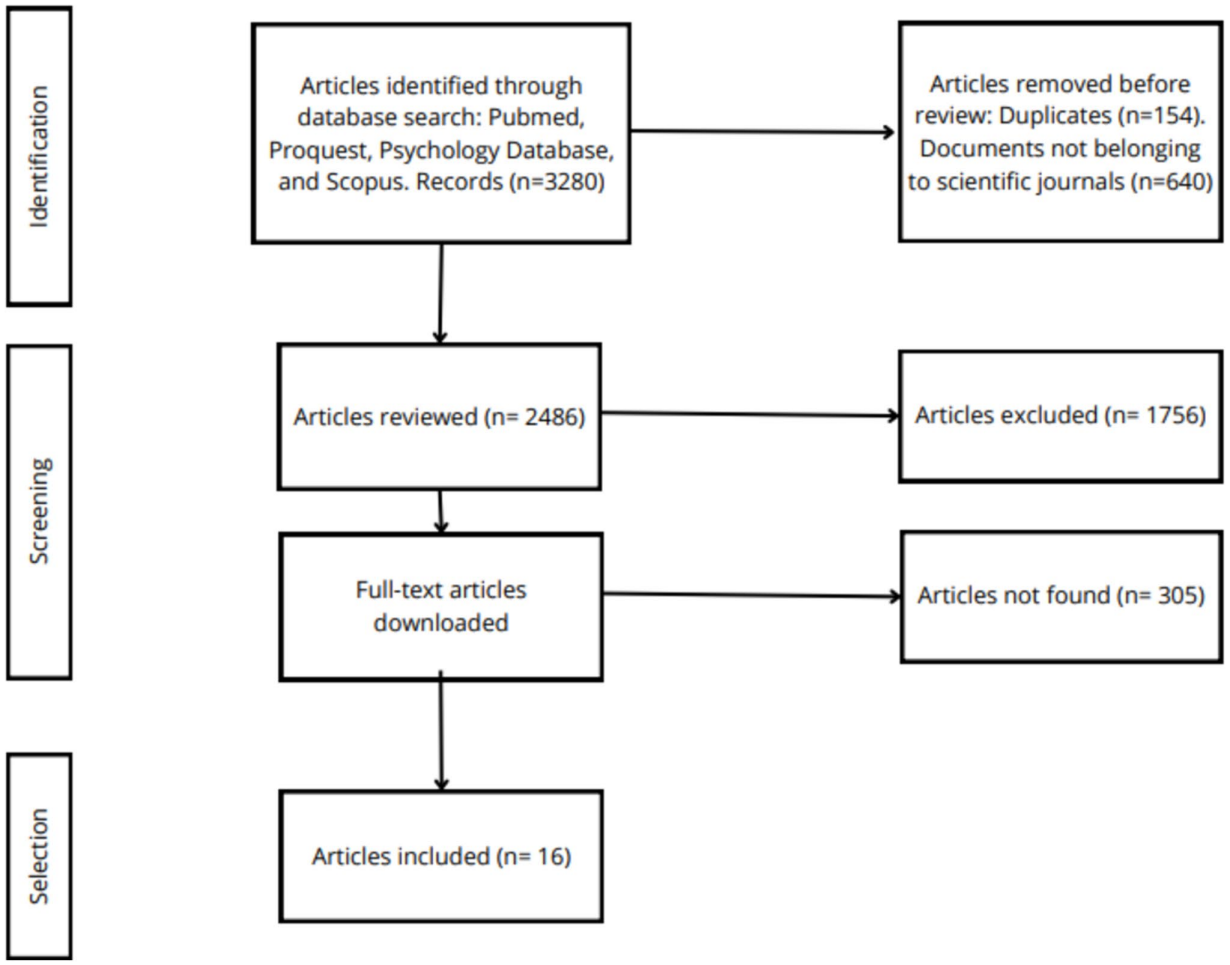
PRISMA diagram.

Out of the 3,280 articles (see Figure 1), 1,756 were excluded for not meeting the aforementioned inclusion criteria. Additionally, 305 articles were excluded because they were not available in full text. Finally, the review of potential articles through title and abstract screening led to the selection of 16 articles for analysis in the present study (see Table 1).

**Table 1:** Studies on TAA in patients in palliative care.

Author	Type of study	Number of sessions and duration	Sample	Professionals	Type of therapy	Results
Bouchard et al. (2004)	Pilot project	53 sessions 1 year	27 children aged 3 to 16 years	Nurses	Dog therapy	Contact with an animal increases the feeling of physical and emotional well-being.
Gagnon et al. (2004)	Descriptive design	39 sessions over 12 months	16 parents and 12 nurses	Experts consisting of 3 nurses and a professor of measurement and evaluation	Dog therapy	AAT can help alleviate patients’ psychological distress.
Orlandi et al. (2007)	Experimental study	25 weeks	89 patients	Nurses	Dog therapy	AAT improved the quality of life of patients, reducing their depression and increasing their arterial oxygen saturation.
Buzzini et al. (2009)	Qualitative research	1 session of 30–45 min	6 participants	Researcher	Dog therapy	When patients socialized with the therapy dog, they noticed they forgot they were sick.
Turnbach (2014)	Within-subjects design study	4 sessions over 3 weeks	200 patients	Nurses and researcher	Dog therapy	Therapy dogs have a positive impact on patients and other people.
Alery (2015)	Qualitative case study	4 sessions, over 6 weeks	1 palliative care patient and their family	Researcher	Dog therapy	AAT is beneficial for the patient and the family.
Coleman (2016)	Qualitative research	10 sessions	10 participants	Palliative care professionals	Dog therapy	Patients who receive AAT appear happier.
Moreira et al. (2016)	Qualitative research	4 visits	16 participants	Healthcare professionals, dog handler, and researchers	Dog therapy	The practice is beneficial for patients and promotes their health.
Schmitz et al. (2017)	Qualitative content analysis	84 sessions over 12 weeks	52 patients	Therapy companion dog teams and their respective caregivers	Dog therapy	AAT can be a valuable and practicable complement to the interdisciplinary therapeutic repertoire of palliative care in the hospital setting.
Krause-Parello et al. (2018)	Cross-sectional study	Experimental session of 20 min	25 patients	Dog handler and clinical psychologist	Dog therapy	AAT had a measurable impact on salivary cortisol levels and heart rate.
Cowfer et al. (2021)	Qualitative cross-sectional study	12 weeks	9 children and 12 parents	Trainer, medical team, and research team	Dog therapy	Patients and their families perceive AAT as beneficial and require few changes.
Quintal and Reis-Pina (2021)	Single case study	Weekends (duration not specified)	1 patient	Medical health team and researcher	Dog therapy	The patient showed a reduction in symptoms of pain, dyspnea, anxiety, depression, fatigue, and drowsiness.
Silva et al. (2021)	Quasi-experimental study	1 session each morning per week, from March to December	80 participants	Certified professionals with formal training in AAT	Dog therapy	A session of AAT was beneficial for reducing pain, depression, and emotional distress.
Cairns et al. (2022)	Exploratory mixed methods study	4 visits over 2 weeks	6 patients aged 58 to 82 hospitalized in a provincial palliative care center	Nurse and horse handling staff	Equine therapy	The offered equine therapy helped patients satisfy their need to live in the moment while suffering from a terminal illness.
López-Fernández et al. (2024)	Prospective, quasi-experimental, and non-randomized study	2 sessions of 45 min each day, for 12 months	61 patients	Occupational therapist, psychologist, and therapy dog	Dog therapy	AAT is feasible, safe, and highly accepted.
Mahoney et al. (2024)	Randomized controlled trial	2–11 sessions over 12 weeks	19 children and 21 parents	Research team and medical team	Dog therapy	AAT can reduce caregivers’ anxiety.

## Results

Firstly, regarding the impact of Animal-Assisted Therapy (AAT) on different aspects of a person’s life in palliative care, several studies have been conducted. Silva et al. (2021) demonstrated that a single session of dog-assisted therapy was beneficial in reducing pain, emotional distress, and feelings of depression. The results also suggested potential benefits in reducing anxiety and anger. According to Turnbach (2014) study, society benefits from knowing that their loved ones in Palliative Care are allowed to have a canine companion, which can enhance the quality of life for many patients by reducing their own stress, the stress of staff, and family members.

Another study by Silva and Osório (2018) determined that AAT has a positive impact on various psychological and physiological variables, such as better adaptation to the hospital environment, reduced stress, anxiety, depressive symptoms, and cortisol levels. It has also been shown that visual communication and touching animals can trigger the release of various substances in the human body, including oxytocin, endorphins, and serotonin, which can reduce pain, anxiety, and stress, while increasing feelings of pleasure and relaxation. Increased communication and social relationships have also been observed.

Additionally, Bouchard et al. (2004) highlighted that having an animal by the bedside of a sick person helps alleviate anxiety, loneliness, boredom, and can reduce depressive states. Contact with an animal increases the sense of physical and emotional well-being, develops a sense of normalcy and being essential to another being, and provides affection and attention. Moreira et al. (2016) further explained that AAT can reduce anxiety and stress, promote relaxation, decrease loneliness and isolation, soften the heavy hospital environment, improve interpersonal relationships, and enhance communication between healthcare teams and patients.

Krause-Parello et al. (2018) collected data on blood pressure, heart rate, and salivary biomarkers—cortisol, alpha-amylase, and immunoglobulin A—before, immediately after, and thirty minutes post-experimental and control conditions. They observed significant decreases in cortisol levels from the pre-time period to thirty minutes post-experiment. Additionally, they noted a significant reduction in heart rate under the same conditions.

Other research has indicated improvements in anxiety and stress, along with increased socialization and reduced stress, improved mood, increased self-awareness, and a greater sense of control. In terms of physical benefits, there has been a decrease in blood pressure, pain, fatigue, and an increase in appetite (Cairns et al., 2022).

Regarding the second objective on the experiences and opinions of patients, families, and healthcare professionals, Cowfer et al. (2021) reported that nearly all participants highlighted positive aspects, such as enjoyment or benefits from participating in therapies. Both parents and medical staff felt that interactions between patients and animals in the hospital were favorable and beneficial. In Quintal and Reis-Pina’s study (Quintal and Reis-Pina, 2021), the medical health team noted a shift in the prescribed rescue therapy for the patient, addressing both pain and dyspnea during animal visits.

Coleman (2016) results showed that patients receiving AAT in Palliative Care expressed feeling happier, more relaxed, and more communicative. Alery (2015) reported positive responses from family members of patients towards AAT, indicating that therapies were meaningful for the patient and improved their quality of life. Bouchard et al. (2004) also noted several positive aspects of therapy, reporting that in the presence of a therapy dog, children gained self-confidence, developed a friendship with the animal, and as a result, were happier. Nurses agreed that dog visits promoted children’s adaptation and helped them recover after other treatments.

Regarding the last objective, Schmitz et al. (2017) determined that while there are several Palliative Care centers offering these therapies, there is still a scarcity of scientific research demonstrating their effectiveness and feasibility. However, various studies have shown that the use of therapy dogs in Palliative Care can significantly improve patient well-being. Mahoney et al. (2024) demonstrated the feasibility of using AAT in children with advanced cancer. Although more research is needed to determine the effectiveness of AAT in pediatric patients with advanced cancer and their caregivers, the results are promising in terms of reducing caregiver anxiety.

López-Fernández et al. (2024) showed in their study that implementing an AAT project is feasible, safe, and highly accepted among participants and healthcare staff. AAT is effective in reducing pain, fear, and anxiety, and thus could be considered a complement to non-pharmacological therapy. Cowfer et al. (2021) emphasized the need for further studies to fully evaluate the effectiveness and feasibility of AAT in Palliative Care.

## Discussion

### Main findings/results of the study

The present study aims to examine the impact of Animal-Assisted Therapy (AAT) on improving the quality of life of individuals in palliative care. To achieve this, the study will analyze its effects on various aspects of patients’ lives, the types of therapies used, the experiences of those who have received these therapies, as well as the efficacy and feasibility of these therapies in the context of Palliative Care.

AAT is an innovative therapeutic modality that positively impacts various psychological and physiological variables, regardless of the patients and therapeutic context. Several studies have shown that AAT significantly benefits the quality of life of those who receive it. For instance, in 2007, Orlandi et al. (2007) found that including therapy dog visits during chemotherapy reduced anxiety and depression. Later, Quintal and Reis-Pina (2021) demonstrated that pet therapy improved depressive symptoms and significantly enhanced perceived quality of life. AAT can substantially reduce pain, anxiety, depression, and fatigue in cancer patients, as revealed by several studies (Petranek et al., 2018). Additionally, studies on the effect of AAT on patients show a reduction in heart and respiratory rates and a decrease in blood pressure (Quintal and Reis-Pina, 2021).

AAT can be a valuable and feasible complement to the interdisciplinary therapeutic repertoire in hospital-based Palliative Care. Patients in the study conducted by Schmitz et al. (2017) reported positive emotional responses, greater physical relaxation, and increased motivation for physical activity. Additionally, patients receiving AAT are satisfied with the therapy and would recommend it to others.

### What this study adds

Research on AAT in Palliative Care is still in its early stages. However, several recommendations for practice can be established:

1. *Selection of appropriate animals*: It is important to select docile animals trained to interact with patients in this setting.
2. *Proper supervision*: Patients should always be supervised by a qualified professional during sessions.
3. *Adaptation of interventions to individual needs.*
4. *Collaboration with the multidisciplinary team:* AAT should be integrated into the patient’s overall Palliative Care plan and provided by a multidisciplinary team that includes doctors, nurses, social workers, and therapists.

It is crucial to emphasize the importance of this topic for future research, as a greater understanding of these therapies is needed. Some priority areas for research include the mechanisms of action of AAT, the efficacy of AAT for different symptoms, the various types of animals used, the duration and frequency of interventions, and the costs associated with the therapy.

### Strengths and weaknesses/limitations of the study

This work is not without limitations. Firstly, there is a publication bias, as studies with positive results are predominantly published, which can lead to an overestimation of the effect of AAT. Additionally, evaluating the quality of the reviewed articles presents difficulties, as the results can be subjective and hard to measure. Another limitation is the difficulty in finding relevant studies, as AAT is a relatively new field of research. Lastly, the lack of a control group in some studies on AAT in palliative care complicates the determination of whether the observed effects are due to the therapy or other factors.

## Conclusion

Animal-Assisted Therapy (AAT) is an innovative therapeutic modality that has been shown to have a positive impact on various psychological and physiological variables, regardless of the target audience and therapeutic context. For example, reductions in pain, emotional distress, and feelings of depression have been observed. Additionally, AAT is beneficial in reducing anxiety and anger. Participants in these therapies also report high levels of satisfaction, leading to broad acceptance of this strategy by medical teams for managing various medical conditions.

In the context of oncology, AAT is particularly relevant, as the diagnosis and treatment of cancer cause physical and emotional suffering, increasing vulnerability to the development of psychological disorders that can directly affect the patient’s overall clinical condition.

However, there is an urgent need for more studies to thoroughly investigate the potential effects of AAT on palliative care patients. It is advisable to develop clearly formulated therapeutic indications based on specific research in this field. Furthermore, analyzing video-recorded encounters between patients and animals could be of significant scientific interest to describe non-verbal interaction phenomena in detail.

## Data Availability

The raw data supporting the conclusions of this article will be made available by the authors, without undue reservation.
